# Anthropometry, body composition, and athletic performance in specific field tests in Paralympic athletes with different disabilities

**DOI:** 10.1016/j.heliyon.2022.e09023

**Published:** 2022-02-25

**Authors:** Moncef Cherif, Mohamed Ahmed Said, Karim Bannour, Majed M. Alhumaid, Mounira Ben Chaifa, Marwa Khammassi, Abdallah Aouidet

**Affiliations:** a Higher Institute of Sport and Physical Education Ksar-Said, Tunisia; b Department of Physical Education, College of Education, King Faisal University, Al-Ahsa 31982, Saudi Arabia; c Higher Institute of Sport and Physical Education of Kef, Tunisia; d Faculty of Sciences, Bizerte, Tunisia

**Keywords:** Morphological characteristics, Physical and physiological performance, Cerebral palsy, Amputee, Short stature, Intellectual disability

## Abstract

**Purpose:**

The structural appearance of each disabled athlete or the shape of their body, as determined by their individual genotype and influenced by the environment, considerably affects their technical and physical performance. This study sought to examine the morphological characteristics of elite track and field athletes with different disabilities, including their possible effects on physical and physiological performance.

**Methods:**

A total of 66 male elite athletes with cerebral palsy (n = 12), upper arm amputation (n = 12), short stature (n = 20), or intellectual disability (n = 22) were included. For each athlete, height, weight, sitting height, arm span and four skin folds were assessed; ape index, body mass index, body fat percentage, fat mass, fat mass index and fat-free mass values were calculated; and vertical jump, drop jump, countermovement jump, squat jump, repeated sprint ability and Yo-Yo Intermittent Recovery Level 1 tests were performed.

**Results:**

Significant differences were noticed between short stature and the other groups concerning morphological characteristics, however, the best motor performance was observed in amputees and, to a lesser degree, in short stature. In the top performing athletes, physical performance was significantly correlated with body mass index and fat mass index for amputees, and with arm span, ape index, body fat percentage and fat mass index for short stature. Regression analysis revealed that regardless of disability type, physical and physiological performance (except maximum heart rate) were significantly influenced primarily by adipose tissue-specific variables. A significant effect of height, weight, fat free mass, arm span, sitting height, and ape index on drop jump performance with left leg, maximal oxygen consumption, and maximum heart rate was also noticed. The type of disability affects performance in the squat jump and vertical jump tests, and to a lesser extent in the countermovement jump test.

## 1 Introduction

Many studies have reported that individuals with disabilities tend to achieve poorer results on standard fitness tests—in particular, those considering weight and body composition, cardiorespiratory endurance, muscular strength, and agility—relative to their nondisabled peers (Franciosi et al., 2010). It has also been demonstrated that structured exercise regimens may attenuate abnormalities and improve physical fitness (Bauman et al., 2016). It is also evident that the structural appearance of each disabled athlete or the shape of their body, as determined by their individual genotype and influenced by the environment, considerably affects their technical and physical performance (Jaarsma et al., 2014). Thus, the quantification of each athlete's morphological characteristics and the correlation between their body structure and sports performance could later serve as a key element in developing technical and physical abilities (Frontera, 2006). Consequently, somatotyping, or the classification of individuals according to their morphological characteristics, has become a major field of interest for many exercise and sports scientists as well as physiotherapists.

Anthropometry and its effects on athletic prowess have been well documented in able-bodied participants in different types of sports (Kirk, 2016). In many cases, it has been demonstrated that anthropometry could predict success, contributes to a longer sports career, and enhances the motivation and increases the chance of being selected at the elite level, particularly in those sports requiring specific skills or that have unique physical demands (Norton and Olds, 2001). Body proportions in terms of fat mass and fat-free mass have been more widely reported by studies; however, it was found that generalized whole-body measurements are not always an important marker for identifying potential success (Tsukahara et al., 2020). This has prompted researchers to seek out more detailed and precise anthropometric measurements such as certain lengths of body segments, differential growth rates, and distinct indices to reveal more reliable performance indicators (Stratton and Oliver, 2019). Unfortunately, a review of the existing literature shows a lack of information regarding high-performance athletes’ disability development patterns and their relationship with different expressions of the human somatotype.

Given that variable anthropometric requirements in different disabilities would lead to differences in physical and physiological variables, a better understanding of the impact of anthropometric variables on physical and physiological performance and the effects of physiological variables on physical variables among athletes with different disabilities is of greater importance. Thus, the aims of this study were to (1) describe the anthropometrical and body composition profiles and evaluate the physical and physiological performance levels of elite Paralympic track and field athletes with certain characteristics; (2) analyze the between-group differences in anthropometry and physical and physiological performances; and (3) examine the relationships that may exist between anthropometric characteristics and the physical and physiological performance of these athletes.

## 2 Materials and methods

### 2.1 Participants

In all, 66 athletes from the Tunisian Paralympic track and field team volunteered to participate in our study, aged 24.58 ± 3.33 years, and nine of them participated in the 2020 Paralympic games in Tokyo, Japan. All athletes eligible for inclusion compete in elite level sports for at least five years and had participated in the last three tournaments of the Tunisian Para Athletics Championship, with no smoking habit, no sensory or motor deficits, and no ergogenic aid or use of any medication known to affect cardiorespiratory function for six months prior to inclusion in the study. The study was conducted in accordance with the latest declaration of Helsinki (World Medical Association, 2013), and approval was obtained from the local ethics committee of the Tunisian Federation of Sports for the Disabled, Tunisia. All athletes were informed about the study protocol and signed an informed consent form before taking part.

### 2.2 Outcomes and assessments

#### 2.2.1 Weight and body composition

All assessments relating to this research were conducted at the health center of the Mohamed-Kassab Institute of Orthopaedics in La Mannouba, Tunisia; regional training centers for the disabled; and the National Center for Medicine and Sports Sciences in El-Menzah, Tunisia. Before undergoing any testing, each participant was subjected to a clinical examination, including answering a questionnaire reporting their health history and a cardiovascular assessment with electrocardiography, respiratory clinical assessment, and osteoarthritis physical assessment. All anthropometric and body composition measurements were collected by the same expert operators with a minimum of five years of work experience at the Mohamed-Kassab Institute of Orthopaedics. The reliability of measurements was tested as described by Cavedon et al. (2018) and the technical errors of measurements were calculated for each operator; these values were generally less than 1% for the skinfolds and less than 0.5% for the remaining parameters. All measurements were conducted on the right side of the body, following the guidelines of the International Society for the Advancement of Kinanthropometry (Marfell-Jones et al., 2012), and included height, weight, sitting height, arm span, and four skinfolds (biceps, triceps, subscapular, and suprailiac). Height and sitting height were measured using a Harpenden stadiometer (Holtain, Crymych, Wales) with an accuracy of 0.1 cm; skinfold thicknesses were measured with Harpenden skinfold calipers (Baty International, Burgess Hill, England); Arm span was measured from the right fingertips to left fingertips, with the arms extended laterally as far as possible and held parallel to the ground, using a horizontal stadiometer placed behind the athlete (de Lucia et al., 2002); and weight was determined using a platform beam balance (Seca, Hamburg, Germany) with minimal clothing to the nearest 0.02 kg. The sum of the four skinfold measurements was used to estimate body density according to the equation of Durnin and Womersley (1974), as previously reported in Paralympic athletes (Bernardi et al., 2012), and the obtained values were used to calculate athletes’ body fat percentage scores according to Siri (1961). The fat mass was calculated as weight (kg) × body fat percentage and the fat-free mass was calculated as weight (kg) – fat mass (kg). The arm span-to-height ratio, labelled as the “ape index”, was calculated as arm span (m)/height (m); the body mass index (BMI) was calculated as weight (kg)/height squared (m^2^); and the fat mass index was calculated as fat mass (kg)/height squared (m^2^) (Peltz et al., 2010; Lemos et al., 2016; Monson et al., 2018). Participants were prohibited from eating or drinking any beverage for 4 h before testing.

#### 2.2.2 Physical and physiological performance

All tests were performed within two days of the clinical examination in the order described henceforth.

##### 2.2.2.1 Vertical jumping tests

Details of the vertical jumping tests were previously described by Nuzzo et al. (2011) and included the vertical jump, drop jump with the right (DJ-right) or left leg (DJ-left), countermovement jump, and squat jump tests. These tests were performed to evaluate the lower limb muscle characteristics using an Opto-jump dispositive (Microgate SRL, Bolzano, Italy) connected to a personal computer. Three attempts were allowed for each test, with 60 s of rest between trials and 3 min between tests, and the best performance was chosen for analysis. The parameters acquired were the time of flight and height achieved from the center of gravity.

##### 2.2.2.2 Repeated sprint ability test

This test was used to measure the athlete's ability to perform six maximal sprints back and forth along a 15-meter route, interspersed with 30-second episodes of recovery (Turner and Stewart, 2014). Speed was evaluated using two pairs of photocells and reflectors connected to an electronic timer (Tag Hewer, Marin, Switzerland). The photocells were placed at shoulder height and the time was given in hundredths of a second. The photocells were positioned at the start and at the end of a 15-m runway.

##### 2.2.2.3 Yo-Yo Intermittent Recovery Level 1 (YYIR1) test

The protocol of the YYIR1 was previously described by Bangsbo et al. (2008). YYIR1 aims to evaluate athletes' ability to repeatedly perform intense effort and their capacity to rapidly recover from such exercise (Krustrup et al., 2003). Briefly, participants performed two 20-m runs at a gradually increasing speed, interspaced by two 10-second periods of active recovery (5 m each). The test started at a relatively low speed (10–13 km/h) during the first four running bouts, which was then increased by 0.5 km/h after every eight running bouts until exhaustion. The heart rate was also recorded before, during, and after the test using a Polar Sport Tester (Polar, Kempele, Finland). The running speed reached at the point of exhaustion was defined as the participant's maximal aerobic speed (MAS), and the highest heart rate level reached was defined as their maximum heart rate (HRmax).

Oxygen consumption was measured continuously using a wearable portable telemetric device (Cosmed K4b^2^, Rome, Italy), and was considered maximal (VO_2_max.) if 2 of the following 3 criteria were met: (a) the existence of a VO_2_ plateau despite increasing intensity of exercise (change lower than 100 mL/min in the last 30-s stages), (b) a respiratory exchange ratio ≥1.15, and (c) ±10 bpm of age-predicted HRmax, using equation 208 − 0.7 × age to predict the HRmax (Beltz et al., 2016).

### 2.3 Statistical analysis

Using the software G∗Power version 3.1.9.7 (Heinrich Heine University Düsseldorf, Düsseldorf, Germany), and based on the method described by Lakens (2013) and the preliminary data of Bernardi et al. (2010) related to VO_2_max. in Paralympic athletes in various sports, we calculated a minimum sample size of 32 athletes to achieve a standard deviation premeasurement of 5.695 ml/kg/min, while assuming an effect size f = 1.2433, a level of significance α error prob = 0.008, a power (1−β error prob) = 99%, and a number of groups = 4. Considering a dropout rate of 20%, a minimum of 40 athletes should be selected for potential participation in this study at the rate of 10 participants/group.

Data were assessed for normality with the Shapiro-Wilk test and log-transformed where necessary. The between-group comparisons were performed using an analysis of variance (ANOVA), when the ANOVA p-value was significant, a Bonferroni's post-hoc comparison test was performed to determine the differences between groups. The specific correlations between morphological characteristics and physical and physiological parameters were conducted using Pearson's coefficient correlation, applying a Sidak's type I error correction to consider multiple comparisons. The results are represented graphically with a color-coded heat map. The linearity of the predictor variables was tested using scatter plots. The normality and homoscedasticity of the residuals were also verified, and the independent variables were checked for multicollinearity using the variance inflation factor (VIF): a value of VIF exceeding 10 indicated excessive multicollinearity. A principal components analysis was used to handle the collinearity between variables. In addition, a sequential multiple regression procedure was performed to determine the amount of variance in physical and physiological performance that could be explained throw the components derived from the principal components' analysis (Model 1) and the effect of the disability (Model 2). Data were analysed with SPSS version 26 for Windows (IBM Corporation, Armonk, NY, USA), presented as mean ± standard deviation, and the statistical significance was set at α = 0.05.

## 3 Results

### 3.1 Sample characteristics

Participants were classified according to their disability into the following four groups: (1) athletes with cerebral palsy (CP) (disability sport classifications T37–38; n = 12), (2) athletes with upper arm amputation (amputees) (disability sport classifications T46–47; n = 12); (3) athletes with short stature (SS) (disability sport classification F40; n = 20) and (4) athletes with intellectual disability (ID) (disability sport classification T20; n = 22). All the athletes have completed all stages of the experiment.

### 3.2 Outcome data

#### 3.2.1 Anthropometric characteristics

Data related to anthropometric characteristics and physical and physiological performance in disabled elite track and field athletes are shown in Table 1. The between-group comparisons revealed significant differences in height, weight, sitting height, arm span, fat mass, and fat-free mass (p < 0.001 for all) between SS and the other three groups as well as in ape index between SS and ID and CP, respectively (p < 0.001 for both comparisons). Significant differences were also observed in sitting height between CP and ID (p < 0.001); in body fat percentage between CP and ID (p < 0.05); in BMI between SS and ID (p < 0.05), and CP and ID (p < 0.05); and in ape index between SS and CP and ID (p < 0.001 for both comparisons). No significant differences were noted in other comparisons (Table 2).

**Table 1:** Anthropometry, body composition, and physical and physiological performances in elite athletes with different disabilities.

	Cerebral Palsy (N = 12)				Amputee (N = 12)				Short Stature (N = 20)				Intellectual Disability (N = 22)			
	Median	Range	Min.	Max.	Median	Range	Min.	Max.	Median	Range	Min.	Max.	Median	Range	Min.	Max.
Age (years)	26	11.00	20	31	24.00	10	19	29	25.00	10.00	19.00	29.00	23.00	11.00	19.00	30.00
Height (cm)	161	26.00	155	181	163.00	25	153	178	132.50	16.00	125.00	141.00	158.00	27.00	147.00	174.00
Sitting Height (cm)	85.5	12.00	80	92	80.50	25	62	87	64.50	26.00	58.00	84.00	72.00	34.00	60.00	94.00
Arm Span (cm)	168	22.00	155	177	147.00	38	140	178	105.00	37.80	90.20	128.00	162.50	35.00	140.00	175.00
Ape Index	1.0211	0.19	0.92	1.11	0.92	0.26	0.85	1.11	0.79	0.30	0.69	0.99	1.02	0.22	0.86	1.08
Weight (kg)	62.4	20.10	52.7	72.8	60.70	25.3	58	83.3	39.63	25.25	31.25	56.50	66.30	26.00	52.00	78.00
BMI (kg/m^2^)	22.9	8.98	17.66	26.64	23.83	8.71	20.28	28.99	22.56	9.15	19.68	28.83	25.72	10.12	21.87	31.99
BFP (%)	14.36	8.24	11.72	19.96	13.05	9.69	6.64	16.33	12.84	12.49	4.41	16.90	13.89	7.38	11.20	18.58
Fat mass (kg)	9.1024	6.60	7.01	13.61	8.13	8.9	4	12.9	5.39	6.00	1.45	7.45	9.17	3.53	7.25	10.78
FMI (kg/m^2^)	3.2863	3.06	2.19	5.25	3.07	2.79	1.6	4.39	2.98	2.88	.87	3.75	3.61	1.50	2.66	4.16
Fat-Free Mass (kg)	52.875	17.84	45.53	63.37	55.03	21.87	48.53	70.4	34.51	24.08	27.27	51.35	56.80	22.83	44.75	67.58
DJ-Right (cm)	12.5	8.00	9	17	21.00	11	15	26	16.00	5.00	15.00	20.00	12.00	17.00	8.00	25.00
DJ-Left (cm)	10	7.00	8	15	19.00	6	17	23	17.00	3.00	15.00	18.00	11.00	14.00	8.00	22.00
Squat Jump (cm)	16	10.00	14	24	24.50	17	14	31	20.50	9.50	16.50	26.00	15.00	14.00	10.00	24.00
CMJ (cm)	18	13.00	12	25	27.50	16	16	32	21.50	10.00	18.00	28.00	17.00	19.00	10.00	29.00
Vertical Jump (cm)	20.5	10.00	18	28	30.00	18	18	36	25.25	9.00	20.00	29.00	19.50	21.00	11.00	32.00
RSA (m/s)	12.075	3.13	9.55	12.68	9.89	1.9	9.12	11.02	10.43	1.40	10.10	11.50	11.00	3.72	9.75	13.47
MAS (km/h)	12.36	0.85	11.94	12.79	12.68	1.81	12.04	13.85	12.25	.70	11.90	12.60	12.31	2.34	11.83	14.17
VO_2_max (ml/kg/min)	43.33	2.31	42.18	44.49	44.20	4.9	42.47	47.37	43.19	1.51	42.40	43.91	43.19	6.34	41.89	48.23
HRmax. (bpm)	180	30.00	160	190	180.00	20	170	190	186.00	13.00	180.00	193.00	180.00	20.00	170.00	190.00
% recovery	50	11.76	47.06	58.82	55.56	16.71	42.11	58.82	51.50	19.11	42.00	61.11	50.00	25.82	38.89	64.71

Note: BMI, body mass index; BFP, body fat percentage; FMI, fat mass index; DJ, drop jump; CMJ, counter movement jump; RSA, repeated sprint ability; MAS, maximal aerobic speed; VO_2_max, maximal oxygen consumption; HRmax, maximal heart rate.

**Table 2:** Anthropometric characteristics, and physical and physiological performances in elite athletes with different disabilities.

	Cerebral Palsy (N = 12)	Amputee (N = 12)	Short Stature (N = 20)	Intellectual Disability (N = 22)
Age (years)	26 ± 3,43	23,83 ± 3,29	24,85 ± 3,22	23,95 ± 3,34
Standing Height (cm)	165.5 ± 8,33 **c**	162.75 ± 6.63 **c**	133.7 ± 5.57 **a, b, d**	159.23 ± 7.5 **c**
Siting Height (cm)	86,58 ± 4.21 **c, d**	80,25 ± 6.24 **c**	65.7 ± 6.24 **a, b, d**	73,6 ± 10,14 **a, c**
Arm Span (cm)	167,5 ± 6,86 **c**	155,75 ± 16,55 **c**	106,9 ± 9,2 **a, b, d**	160,32 ± 11,3 **c**
Ape Index	1.0135 ± 0.049 **c**	0.886 ± 0.288	0.78 ± 0.069 **a, d**	1.007 ± 0.536 **c**
Weight (kg)	62,50 ± 5,67 **c**	65,36 ± 8,42 **c**	41.83 ± 7,8 **a, b, d**	65,19 ± 7,32 **c**
Body Mass Index (kg/m^2^)	22,91 ± 2,51 **d**	24,65 ± 2,52	23.32 ± 3,43 **d**	25,69 ± 2,05 **a, c**
Body Fat Percentage (%)	14,85 ± 2,52 **c**	12,17 ± 3,18	12,25 ± 3,01 **a**	13,95 ± 1,6
Fat mass (kg)	9,3 ± 1,87 **c**	8,03 ± 2,62 **c**	5,2 ± 1,4 **a, b, d**	9,03 ± 0,96 **c**
Fat Mass Index (kg/m^2^)	3,44 ± 0,87	3,02 ± 0,91	2,85 ± 0,74 **d**	3,57 ± 0,39 **c**
Fat-Free Mass (kg)	53,22 ± 5,06 **c**	57,34 ± 6,9 **c**	36.68 ± 7.22 **a, b, d**	56,15 ± 6,95 **c**
Drop Jump Right (cm)	12,83 ± 2,44 **b, c**	21,25 ± 2,92 **a, c, d**	17.5 ± 1.88 **a, d**	13,59 ± 4,26 **b, c**
Drop Jump Left (cm)	11,08 ± 2,31 **b, c**	19 ± 1,80 **a, d**	17 ± 0,73 **a, d**	12,04 ± 3,67 **b, c**
Squat Jump (cm)	17,58 ± 3,57 **b**	23,5 ± 5,05 **a, d**	21.7 ± 3,18 **d**	16,18 ± 4,71 **b, c**
Counter Movement Jump (cm)	18,91 ± 3,55 **b**	25,5 ± 5,38 **a, d**	22.95 ± 3,75 **d**	17,68 ± 5,32 **b, c**
Vertical Jump (cm)	21,75 ± 3,25 **b**	28,66 ± 5,59 **a, d**	24.9 ± 3.35 **d**	20,04 ± 5,61 **b, c**
Repeated Sprint Ability (m/s)	11.61 ± 1.06 **b, c**	10 ± 0.66 **a, d**	10.4 ± 0.37 **a, d**	11.33 ± 1.1 **b, c**
Maximal Aerobic Speed (km/h)	12,43 ± 0,32	12,76 ± 0,66	12,4 ± 0,21	12,5 ± 0,53
Maximal Oxygen Consumption (ml/kg/min)	43,52 ± 0,88	44,44 ± 1,78	43,32 ± 0.49	43,71 ± 1,45
Maximal Heart Rate (bpm)	177,5 ± 8,66	180 ± 7,38	181 ± 5,13	180,45 ± 6,52
% recovery	51% ± 0,04	53% ± 0,06	51,79% ± 7,66	51% ± 0,7

Results expressed as mean ± SD. a: differs significantly to Cerebral Palsy; b: differs significantly to Amputee; c: differs significantly to Short Stature; d: differs significantly to Intellectual Disability.

#### 3.2.2 Physical and physiological characteristics

The comparison of physical performances revealed significant differences in performance levels in tests of DJ-right, DJ-left, squat jump, countermovement jump, vertical jump, and repeated sprint ability tests in amputees relative to CP (p < 0.001, 0.001, 0.05, 0.01, 0.01, and 0.001, respectively) and ID (p < 0.01 for repeated sprint ability and p < 0.001 for the rest). Significant differences were also observed in all physical variables between SS and ID (p < 0.001 for DJ-left and squat jump and p < 0.01 for the rest); in DJ-right, DJ-left, and repeated sprint ability performance level between SS and CP (p < 0.01, 0.001, and 0.01, respectively) and SS and ID (p < 0.05); and in DJ-right between amputees and SS (p < 0.05). The best motor performance was observed in amputees and SS. No significant difference in physiological performance was found among all groups (Table 2).

#### 3.2.3 Correlation between physical and physiological performance, and anthropometric variables

##### 3.2.3.1 Athletes with cerebral palsy

**Figure 1:**
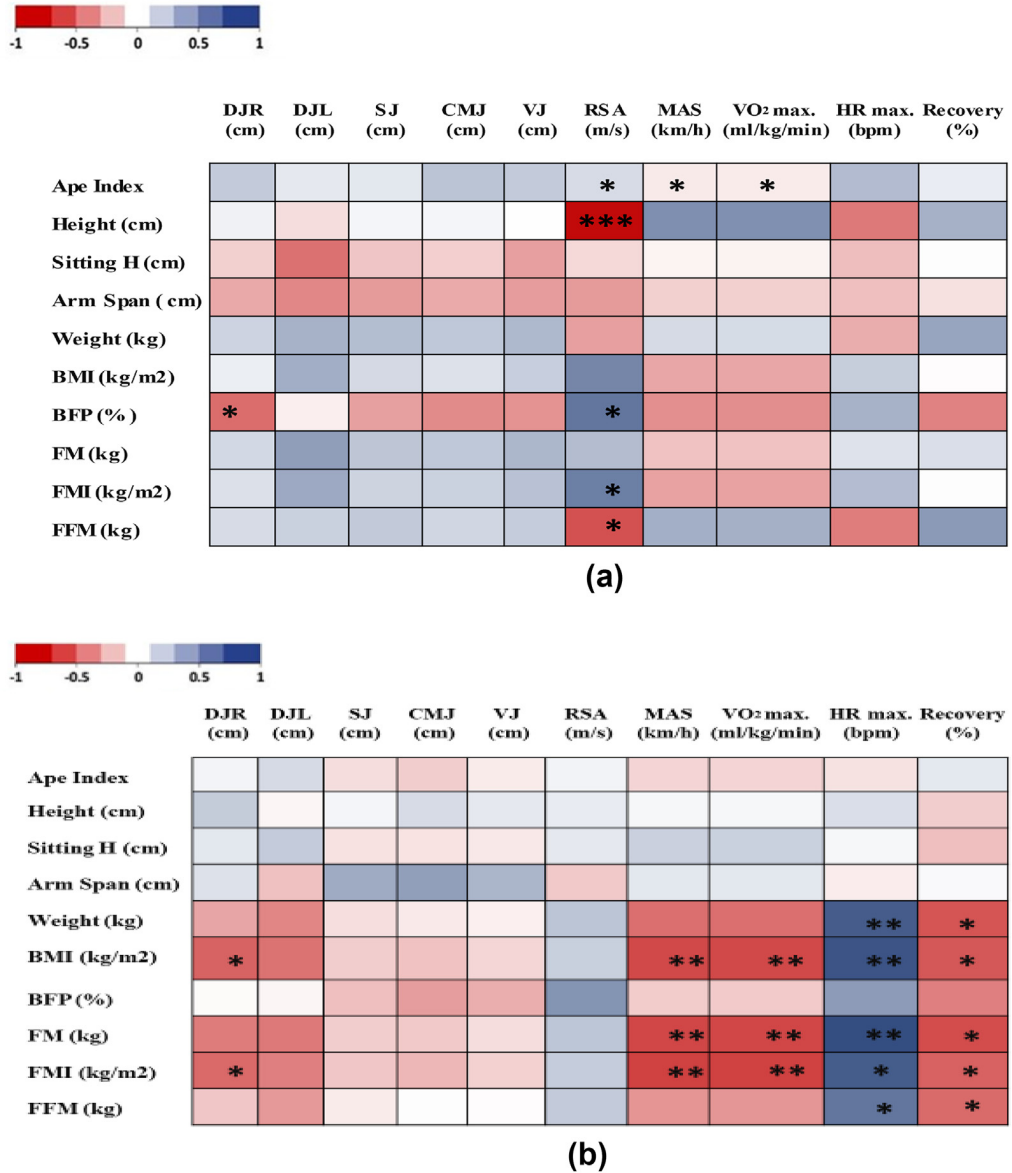
Correlations between the anthropometric characteristics of elite athletes with cerebral palsy (a) or upper limb amputation (b) and their physical and physiological performance. DJR, drop jump right; DJL, drop jump left; SJ, squat jump; CMJ, counter movement jump; VJ, vertical jump; RSA, repeated sprint ability; MAS, maximal aerobic speed; Sitting H, siting height; BMI, body mass index; BFP, body fat percentage; FM, fat mass; FMI, fat mass index; FFM, fat-free mass.

The DJ-right performance was negatively correlated with the body fat percentage (r = −0.576; p < 0.05). A negative correlation was also noticed between the repeated sprint ability performance and the athlete's height (r = −0.959; p < 0.01) and fat-free mass (r = −0.679; p < 0.05). This parameter was also positively correlated with ape index (r = 0.654; p < 0.05), PBF (r = 0.616; p < 0.05), and fat mass index (r = −0.577; p < 0.05; Figure 1a).

##### 3.2.3.2 Amputee athletes

The DJ-right performance depends upon the BMI (r = −0.608; p < 0.05) and fat mass index (r = −0.608; p < 0.05). Negative correlations were noticed between BMI, fat mass, fat mass index, and MAS (r = −0.711, −0.718, and −0.743; p < 0.01, respectively). The BMI, fat mass, and fat mass index affected also the VO_2_max (r = −0.71, −0.718 and −0.742; p < 0.01, respectively), the HRmax (r = 0.767, 0.796 and 0.73; p < 0.01, respectively), and the % recovery (r = −0.651, −0.697, and −0.617; p < 0.05, respectively). In addition, HRmax and % recovery was also correlated with the athlete's weight (r = 0.729 and −0.665; p < 0.01 and 0.05, respectively) and fat-free mass (r = 0.615 and −0.578; p < 0.05, respectively; Figure 1b).

##### 3.2.3.3 Athletes with short stature

**Figure 2:**
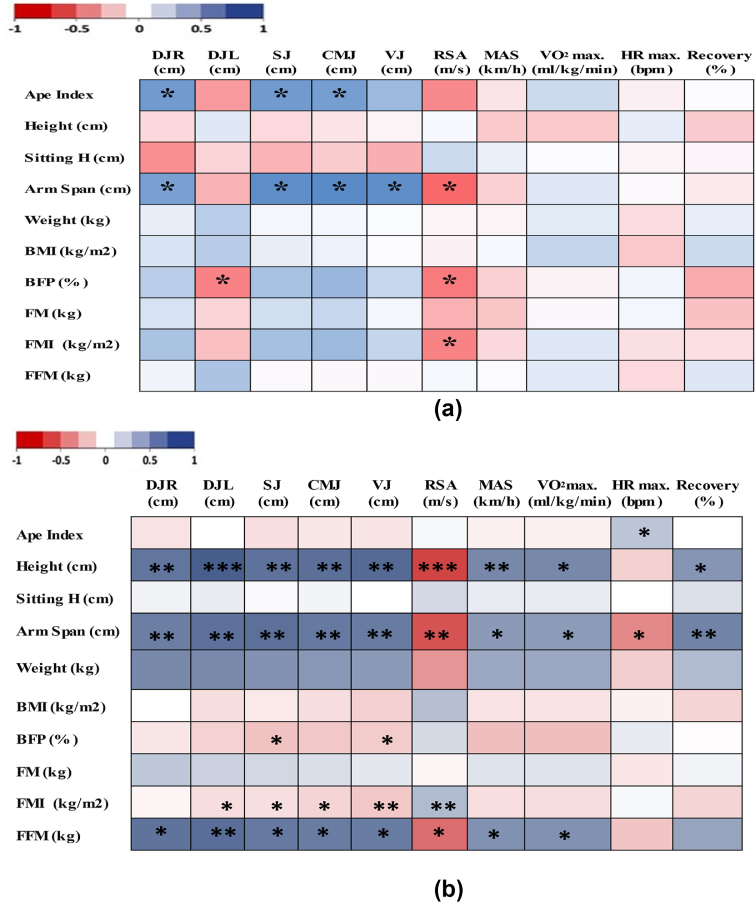
Correlations between the anthropometric characteristics of elite athletes with short stature (a) or intellectual disabilities (b) and their physical and physiological performance. DJR, drop jump right; DJL, drop jump left; SJ, squat jump; CMJ, counter movement jump; VJ, vertical jump; RSA, repeated sprint ability; MAS, maximal aerobic speed; Sitting H, siting height; BMI, body mass index; BFP, body fat percentage; FM, fat mass; FMI, fat mass index; FFM, fat-free *mass.*

Pearson's correlation coefficient revealed that the ape index was positively correlated with results of the DJ-right (r = 0.476; p < 0.05), squat jump (r = 0.449; p < 0.01), and countermovement jump (r = 0.454; p < 0.05) tests. A significant correlation was also noticed between the arm span and performance level during the DJ-right (r = 0.447; p < 0.05), squat jump (r = 0.535; p < 0.05), countermovement jump (r = 0.546; p < 0.05), vertical jump (r = 0.523; p < 0.05), and repeated sprint ability (r = −0.532; p < 0.05) tests. However, the DJ-left, and repeated sprint ability were negatively correlated with the body fat percentage outcome (r = -0.425 and -0.458; p < 0.05, respectively), and with the fat mass index for the repeated sprint ability only (r = -452; p < 0.05; Figure 2a).

##### 3.2.3.4 Athletes with intellectual disability

Analysis revealed significant relationships between all physical and physiological performance levels and athlete height, except for the HRmax; fat-free mass, except for the HRmax and the % recovery; and the arm span. Level of performance during the DJ-left (r = -0.454; p < 0.05), squat jump (r = -0.436; p < 0.05), countermovement jump (r = -0.514; p < 0.05), vertical jump (r = -0.568; p < 0.01) and repeated sprint ability (r = 589; p < 0.01) tests was also significantly correlated with BMI. In addition, the squat jump and vertical jump were negatively correlated with the body fat percentage (r = -0.454 and -0.436; p < 0.05, respectively; Figure 2b).

#### 3.2.4 Associations between morphological characteristics and physical and physiological performance

The partial component analysis was carried out with 1 as the minimum eigenvalue of the factors. After Varimax rotation with Kaiser normalization, a three-factor solution was extracted. Factor 1 includes height, weight, fat-free mass, arm span, sitting height, and ape index; Factor 2 includes body fat percentage, fat mass, and fat mass index; and factor 3 includes age and BMI (Table 3). The regression analysis showed that, regardless of the type of disability, physical and physiological performance (except HRmax) was significantly affected mainly by the second factor which includes the adipose tissue specific variables. A significant effect of the first factor on the performance at the DJ-left, MAS, VO_2_max and HRmax tests was also noticed. The proportion of variability explained by these factors, together or separately, ranged from 9.4% to 23.1%. A negative effect of the type of disability (CP as ref.) was observed on the performance at the squat jump test (R^2^ changes from 9.5% to 16.8%), and that at the vertical jump (R^2^ changes from 10.8% to 18%). A large, but statistically not significant, effect was also observed on the performance at the countermove jump test (R^2^ changes from 10% to 16.4%). No significant effects of the type of disability were noticed on the other physical and physiological performances (Table 4).

**Table 3:** Factors extracted, eigenvalues and proportion of variance after rotation.

Components	1	2	3
Height	**0.965**	0.028	0.007
Weight	**0.841**	0.222	0.446
Age	-0.063	0.184	**-0.449**
Body Mass Index	0.111	0.390	**0.853**
Fat Free Mass	**0.839**	0.097	0.484
Percentage Body Fat	0.134	**0.969**	0.189
Fat Mass	0.078	**0.937**	-0.252
Fat Mass Index	0.617	**0.749**	0.155
Arm Span	**0.951**	0.133	0.069
Sitting Height	**0.720**	0.096	-0.412
Arm Span to Height	**0.791**	0.216	0.118
Eigenvalues	50.72	20.03	10.43
% of variance after rotation	43.74	24.52	15.21

Extraction Method: Principal Component Analysis. Rotation Method: Varimax with Kaiser Normalization. Retained eigenvalue of the factors >1.

**Table 4:** Sequential multiple regression models for associations between morphological characteristics and physical and physiological performance of elite athletes with different disabilities.

	Model		Beta-coefficient	Std. Error	t	Sig.	R^2^
Drop Jump Right (cm)	1	Constant	15.879	0.512	30.99	0.001	0.109
		REGR factor score 2	−1.35	0.516	−2.615	0.011	
	2	Constant	18.316	1.573	11.645	0.001	0.146
		REGR factor score 2	−1.296	0.511	−2.538	0.014	
		Disability	−0.874	0.534	−1.637	**NS**	
Drop Jump Left (cm)	1	Constant	14.57	0.451	32.296	0.001	0.174
		REGR factor score 1	−0.961	0.455	−2.114	0.039	
		REGR factor score 2	−1.306	0.455	−2.873	0.006	
	2	Constant	16.773	1.383	12.126	0.001	0.211
		REGR factor score 1	−1.202	0.47	−2.556	0.013	
		REGR factor score 2	−1.257	0.449	−2.799	0.007	
		Disability	−0.79	0.47	−1.682	**NS**	
Squat Jump (cm)	1	Constant	19.27	0.595	32.411	0.001	0.095
		REGR factor score 2	−1.475	0.599	−2.461	0.017	
	2	Constant	23.194	1.788	12.974	0.001	0.168
		REGR factor score 2	−1.387	0.58	−2.39	0.02	
		Disability	−1.408	0.607	−2.318	0.024	
Counter Movement Jump (cm)	1	Constant	20.833	0.643	32.41	0.001	0.10
		REGR factor score 2	−1.686	0.648	−2.603	0.012	
	2	Constant	24.502	1.954	12.538	0.001	0.164
		REGR factor score 2	−1.604	0.634	−2.529	0.014	
		Disability	−1.316	0.664	−1.983	**NS**	
Vertical Jump (cm)	1	Constant	23.379	0.654	35.734	0.001	0.108
		REGR factor score 2	−1.808	0.659	−2.742	0.008	
	2	Constant	27.697	1.967	14.08	0.001	0.180
		REGR factor score 2	−1.712	0.639	−2.681	0.009	
		Disability	−1.549	0.668	−2.318	0.024	
Repeated Sprint Ability (seconds)	1	Constant	10.891	0.115	95.112	0.001	0.221
		REGR factor score 2	0.482	0.115	4.174	0.001	
	2	Constant	10.776	0.359	30.032	0.001	0.222
		REGR factor score 2	0.479	0.116	4.113	0.001	
		Disability	0.041	0.122	0.338	**NS**	
Maximal Aerobic Speed (km/h)	1	Constant	12.481	0.053	234.666	0.001	0.194
		REGR factor score 1	0.159	0.054	2.961	0.004	
		REGR factor score 2	−0.129	0.054	−2.403	0.019	
	2	Constant	12.352	0.166	74.452	0.001	0.202
		REGR factor score 1	0.173	0.056	3.062	0.003	
		REGR factor score 2	−0.132	0.054	−2.445	0.017	
		Disability	0.046	0.056	0.82	**NS**	
VO_2_ max. (ml/kg/min)	1	Constant	43.685	0.143	305.215	0.001	0.176
		REGR factor score 1	0.398	0.144	2.757	0.008	
		REGR factor score 2	−0.337	0.144	−2.334	0.023	
	2	Constant	43.377	0.447	97.042	0.001	0.183
		REGR factor score 1	0.431	0.152	2.838	0.006	
		REGR factor score 2	−0.344	0.145	−2.368	0.021	
		Disability	0.111	0.152	0.729	**NS**	
Maximal Heart Rate (bpm)	1	Constant	181.348	0.785	230.882	0.001	0.231
		REGR factor score 1	−3.198	0.791	−4.041	0.001	
	2	Constant	180.968	2.463	73.472	0.001	0.231
		REGR factor score 1	−3.157	0.838	−3.769	0.001	
		Disability	0.137	0.837	0.163	**NS**	
Recuperation (%)	1	Constant	51.817	0.79	65.551	0.001	0.094
		REGR factor score 2	−1.912	0.797	−2.4	0.019	
	2	Constant	52.072	2.479	21.004	0.001	0.094
		REGR factor score 2	−1.906	0.805	−2.369	0.021	
		Disability	−0.091	0.842	−0.108	**NS**	

**Note:** Model 1: tests the amount of variance that could be explained by the components derived from the partial component analysis; Model 2: tests the effect of the type of disability on physical and physiological performance. NS: not significant.

## 4 Discussion

Studies that focus on sports participation among people with disabilities are limited due to the limited total population of disabled athletes and the enormous variability in disabilities within the population itself, resulting in a wide territorial distribution for most studies (Cavedon et al., 2018). One main finding of this study was that mean BMIs in track and field athletes with intellectual disabilities was 25,69 kg m^−2^, which are above the current cutoff values for an overweight status (Fox et al., 2014). SS also have the smallest body dimensions, together with reduced fat proportions, and, yet, together amputee athletes, they demonstrated the greatest physical performance. Additionally, the correlation analysis noted that, in SS, the DJ-right, squat jump, countermovement jump, vertical jump, and repeated sprint ability performance levels were significantly correlated with arm span; conversely, in amputees, leg strength and power depend upon mainly the BMI and fat mass index. Significant relationships were also observed between physical and physiological performance and height, fat-free mass, and arm span in ID and in DJ-right vs. body fat percentage, in repeated sprint ability vs. ape index, height, body fat percentage, fat mass index and fat-free mass, and in ape index vs. MAS and VO_2_max. in athletes with cerebral palsy, respectively. The regression analysis showed that, in all participants, physical and physiological performance (except HRmax) was significantly affected by the body fat percentage, fat mass and fat mass index. A significant effect of height, weight, fat-free mass, arm span, sitting height, and ape index on the performance at the DJ-left, MAS, VO_2_max and HRmax tests was also noticed. A negative effect of the type of disability was observed on performances at squat jump and vertical jump tests.

Our findings are in line with those of previous studies indicating that favorable anthropometric characteristics lead to exceptional biomechanical and physical efficiency in selected sports. In fact, several reports have suggested that morphological characteristics are important determinants in many sports. Certain body composition parameters, mainly those related to fatty mass, can significantly influence both physical and physiological performance (Maciejczyk et al., 2014). According to Carter (1996) athletes' unique body structure and morphological characteristics could explain 25%–60% of the variance in physical fitness test results. He affirmed in addition that the most successful athletes have the appropriate characteristics for the sports-related tasks they face; therefore, studying the different links between these characteristics and tasks will improve our understanding of the aspects of human physics. An athlete's weight, fat mass, and muscle mass all appear to be important factors, particularly influencing their jumping performance, MAS, VO_2_max, and running speed ability. It is accepted that lower body fat is associated with greater muscle mass, which would give athletes an advantage in both jumping and running. Greater fat-free mass would imply greater economy in the movement of the weight both vertically and horizontally; however, fat mass is an extra load acting as a dead weight that must be moved (Nikolaidis et al., 2015). A negative correlation was reported by Apostolidis et al. (2004) between body fat percentage and performance time in running speed and high-intensity shuttle runs among basketball players.

Anthropometric studies have largely suggested that the choice of large athletes in terms of height, weight, and BMI appears as a criterion of success that can in some ways promote access to the sports' practice among disabled athletes (Temple et al., 2010). For this reason, most coaches today gave considerable importance to the evaluation of disabled athletes' morphological potential to ensure a proportional orientation to the selected physical activity and achieve success (Wu et al., 2010). Nevertheless, our results showed that the SS performed as well as amputees and significantly better than both CP and ID, although they had significantly lower longitudinal measurements, with high fat-free mass and low fat-mass values. We also noticed that amputees performed better than CP or ID, although they present almost the same anthropometric characteristics, indicating that the nature of an athlete's disability affects their level of physical performance. In fact, athletes are classified or categorized by their degree of impairment to ensure equitable competition. For example, athletes with physical disabilities such as amputation are evaluated and placed in a sports competition with a specific classification. During competitions, many sports such as basketball and table tennis use functional or integrated systems, which allow athletes with a variety of disabilities to compete with one another. Some sports such as track and field events rely on disability-specific classification systems that evaluate both the athlete's function and etiology of their disability.

One measurement that has been identified as being of greater concern is the relationship between the arm span, ape index, and physical and physiological performance levels. The most performant athletes are those with the smallest arm span-to-height ratio. Values recorded in amputees and SS are below the average ratio of 1:1, which is the ratio perceived in the general human population (Kirk, 2016). Our findings disagree with those of Cavedon et al. (2018), who noted that an arm span larger than the height could offer some advantage in some sports and, therefore, could be a selective criterion; this is especially the case within wheelchair basketball and water polo, where the most performant players are found to have greater arm span length than height (Lozovina et al., 2009). In contrast, this parameter was found to have no effect in certain other sports, like climbing (Mermier et al., 2000) and cricket bowling (b). However, Kirk (2016) reported that several elite mixed martial arts competitors were ranked higher or had competed for/won world titles while having smaller arm span values. This can be explained by the relatively large amounts of time spent in grappling movements or in a clinch, making any differences in arm span and/or ape index largely meaningless. In addition, it was demonstrated that competitors of a shorter stature with smaller arm span values are characterized with a higher potential for speed and quicker reaction times, giving them a natural advantage (Kirk, 2016).

Physical activity is widely accepted as a necessary component for individual health. In recent years, an increasing emphasis on the role of sports and physical activity in enhancing health and quality of life among individuals with disabilities and chronic illness has emerged (Golubovic et al., 2012; Khammassi et al., 2020). Athletes with disabilities can generally receive the same health benefits from exercise and sports training as their able-bodied counterparts. Giacobbi et al. (2008) examined links between physical activity and the quality of life experienced by individuals with physical disabilities recruited from a wheelchair user's basketball tournament, reporting that individuals who use wheelchairs perceived several psychological, social, and health benefits associated with physical activity involvement. These findings support the idea that physical activity is important to support self-efficacy beliefs, feelings of empowerment, and motivation for continued involvement among individuals with physical disabilities.

Evidence suggests also that certain factors, including body fat, muscle mass, and physical variables, significantly influence physiological performance (Elmahgoub et al., 2009). In fact, disabled athletes still appear very active, even conducting the practice of ordinary physical activities like nondisabled athletes at high levels. As such, the comparison of physiological responses in concert with VO_2_max, HRmax, and % recovery values revealed no significant difference between the study groups. Participation in intensive training has also been shown similarly to benefit individuals with neuromuscular impairments of cerebral origin (Richter et al., 1996).

During the last decade, sports for athletes with disabilities have moved away from incorporating a medical rehabilitation model and toward a competitive sports model. The relationship between sports and rehabilitation, however, continues to have relevance. The merit of this study includes anthropometric measurements of elite sportsmen with different disability sports codes that have not previously been investigated to this scale. The design of the study allowed us to evaluate the effects of anthropometric characteristics in physical performance; in fact, using Pearson's correlation, no significant relationship was observed between morphological characteristics and anaerobic performance in athletes with short stature. Sports participation enhances mental health in a variety of ways (Fernhall, 1993). There are several barriers to elite athletes accessing help for mental health concerns. Competitive athletes may have fewer positive attitudes toward seeking help for mental health problems than nonathletes, perhaps partially due to such being perceived as a weakness (Bauman et al., 2016). Regular participation in sports and physical activity enhances mental health and well-being, improves physical health, reduces symptoms of anxiety and depression, increases self-esteem and confidence, and lessens the risk of developing serious physical health conditions (Wu et al., 2010).

### 4.1 Strengths and limitations

To our knowledge, this is the first study that has sought to examine the morphological characteristics of elite track and field athletes with different disabilities (CP, Amputees, SS, and ID), together their possible effects on physical and physiological performance. We were able to assess a wide variety of anthropometric, physical, and physiological characteristics specific to each group of elite athletes. Moreover, we were able to analyze the different relationships that may exist between all the variables, and thus to provide useful information to athletes and their coaches to better equip them to test their personal limits, improve training methods, and pursue their dreams and sporting goals (Van de Vliet et al., 2006). However, the current results should be interpreted with caution due to the presence of some limitations. First, due to a lack of standard assessment methods for athletes with disabilities, specific predictive equations for able-bodied peers were used, which could bias the results (Rodrigues et al., 2018). Second, the group sizes were relatively small due to the limited number of participants in these sports. Therefore, descriptive data for each group of athletes may not be generalizable to all elite athletes with the same disability. Third, the present study did not assess the relationship between the strength of athletes' upper limbs and their anthropometric characteristics. Since three sports specialties of 24 athletes mainly used upper extremities (discus throw, shot put, javelin), it is possible that the performance achieved in such physical quality correlated with the anthropometric characteristics of these athletes. Future studies should investigate the relationship between anthropometric characteristics and measures of general strength as well as upper limb strength, primarily in throwers, to identify the best predictors of performance in Paralympic track and field athletes. Finally, no female athletes were evaluated in this study, so the findings can only be generalized to male athletes. Further studies in this field of more athletes of both sexes are needed to build a strong database for elite Paralympic sports (Bernardi et al., 2010).

## 5 Conclusions

The current study demonstrates that certain morphological characteristics can enhance physical performances in track and field athletes with different disabilities, as has already been demonstrated by other authors (Zwierzchowska et al., 2015; Temple et al., 2010). Data noted that the best motor performance was observed in the amputee athlete group and, to a lesser degree, in the SS group, with similar physiological performances between the four groups. However, significant differences were noticed between SS and the other three groups concerning height, weight, sitting height, arm span, fat mass, and fat-free mass, and between SS and CP and ID athletes concerning ape index. Significant differences were also observed between CP and SS in body fat percentage and between SS and ID athletes in BMI and fat mass index. No significant differences in anthropometric characteristics were noted in amputees compared to CP and ID. In the top performing athletes, physical performance was significantly correlated with BMI and fat mass index for amputees, and with arm span, ape index, body fat percentage and fat mass index for SS. Regression analysis revealed that, regardless of the type of disability, physical and physiological performance (except HRmax) was significantly influenced primarily by adipose tissue-specific variables. The type of disability affects performance at squat jump and vertical jump tests, and to a lesser extent at the countermovement jump test.

Referring to previous data, we observed that the results recorded, especially during the jump tests, are not far from those of so-called “valid sportsmen” (Bauman et al., 2016), and this may lead to a positive integration of similar training programs for athletes in similar disciplines. Our results also demonstrated that with adequate training and sufficient practice time, most athletes with different disabilities can compete successfully with or against many of their able-bodied peers. Finally, the results of this study can also be used to re-evaluate initiatives such as the Special Olympics.

## Declarations

### Author contribution statement

Moncef Cherif, Karim Bannour and Mounira Ben Chaifa: Conceived and designed the experiments; Performed the experiments; Contributed reagents, materials, analysis tools or data.

Abdallah Aouidet: Conceived and designed the experiments; Analyzed and interpreted the data; Contributed reagents, materials, analysis tools or data; Wrote the paper.

Majed M. Alhumaid: Conceived and designed the experiments; Contributed reagents, materials, analysis tools or data; Wrote the paper.

Mohamed Ahmed Said: Analyzed and interpreted the data; Contributed reagents, materials, analysis tools or data; Wrote the paper.

Marwa Khammassi: Performed the experiments; Analyzed and interpreted the data; Contributed reagents, materials, analysis tools or data; Wrote the paper.

### Funding statement

This research did not receive any specific grant from funding agencies in the public, commercial, or not-for-profit sectors.

### Data availability statement

Data will be made available on request.

### Declaration of interests statement

The authors declare no conflict of interest.

### Additional information

No additional information is available for this paper.
